# New Appliances and Things Medical

**Published:** 1900-06-09

**Authors:** 


### NEW APPLIANCES AND THINGS MEDICAL.

[We shall be glad to receive, at our Office, 28 & 29, Southampton Street, Strand, London, W.C., from the manufacturers, specimens of all new preparations and appliances which may be brought out from time to time.]

### SCOTCH WHISKY.

(DANIEL CRAWFORD, 81, QUEEN STREET, GLASGOW.)

WE have received a sample of the above whisky, and after careful examination are able to pronounce it a fine and well-matured spirit, with an exceeding pleasant and mellow flavour. Every precaution is taken by the vendors to prevent fraud or tampering with their labels, bottles, or contents, and consequently customers ordering Crawford's whisky from retailers may feel confident that they are obtaining the genuine article.

